# Supplementary material for: Patterns of Intron Gain and Loss in Fungi
Source: PLoS Biol. 2004 Nov 30;2(12):e422. doi: 10.1371/journal.pbio.0020422 (PMC532390; doi:10.1371/journal.pbio.0020422)
Supplement: Table S1 — Also available at http://genes.mit.edu/NielsenEtAl/. (4.3 MB ZIP). [file pbio.0020422.st001.zip › NielsenEtAl/html/432.html]

AN4563.1.NCU00685.1.MG02829.1.FG08731.1


```
 CLUSTAL W (1.82) Multiple Sequence Alignments - Introns Inserted


Sequence 1: MG02829.1	338 aa
Sequence 2: FG08731.1	371 aa
Sequence 3: NCU00685.1	334 aa
Sequence 4: AN4563.1	363 aa
Alignment Length: 393 aa
Number Identitical Residues: 281 aa
Alignment Score (without introns) 10975


MG02829.1 	MCICIWQ~DLRVGNKYRIGRKIGSGSFGDIYLGTNIISGE~EIAIKLESVKAKHPQLEYE
NCU00685.1	---MTTM0DLRVGNKYRIGRKIGSGSFGDIYLGTNIISGE~EIAIKLESVKAKHPQLEYE
FG08731.1 	---MTTM0DLRVGNKYRIGRKIGSGSFGDIYLGTNIISGE~EIAIKLESVKAKHPQLEYE
AN4563.1  	------M0DLRVGNKYRIGRKIGSGSFGDIYLG-----AF0EIAIKLESVKAKHPQLEYE
          	        *************************     .  *******************

MG02829.1 	ARVYKSLAGGVGIPFVRWFGTECDYNAMVLDLLGPSLEDLFNFCNRKFSLKTVLLLADQL
NCU00685.1	ARVYKSLAGGVGIPFVRWFGTECDYNAMVLDLLGPSLEDLFNFCNRKFSLKTVLLLADQL
FG08731.1 	ARVYKSLAGGVGIPFVRWFGTECDYNAMVLDLLGPSLEDLFNFCNRKFSLKTVLLLADQL
AN4563.1  	ARVYKSLAGGVGIPFVRWFGTECDYNAMVIDLLGPSLEDLFNFCNRKFSLKTVLLLADQL
          	*****************************:******************************

MG02829.1 	ISRIEYIHAKSFIHRDIKPDNFLMGIGKRGNQVNVIDFGLAKKYRDPKTHFHIPYRENKN
NCU00685.1	ISRIEYIHAKSFIHRDIKPDNFLMGIGKRGNQVNVIDFGLAKKYRDPKTHFHIPYRENKN
FG08731.1 	ISRIEYIHAKSFIHRDIKPDNFLMGIGKRGNQVNVIDFGLAKKYRDPKTHFHIPYRENKN
AN4563.1  	ISRIEYIHAKSFIHRDIKPDNFLMGIGKRGNQVNVIDFGLAKKYRDPKTHFHIPYRENKN
          	************************************************************

MG02829.1 	LTGTARYASINTHLGVE1QSRRDDMESLGYVMLYFCRGSLPWQGLKAATKKQKYDRIMEK
NCU00685.1	LTGTARYASINTHLGVE~QSRRDDMESLGYVMLYFCRGSLPWQGLKAATKKQKYDRIMEK
FG08731.1 	LTGTARYASINTHLGVE~QSRRDDMESLGYVMLYFCRGSLPWQGLKAATKKQKYDRIMEK
AN4563.1  	LTGTARYASINTHLGVE~QSRRDDMESLGYVMLYFCRGTLPWQGLKAATKKQKYDRIMEK
          	***************** ********************:*********************

MG02829.1 	KMTTPTEVLCRGFPNEFAIYLNYTRSLRFDDKPDYSYLRKIFRDLFVREGFQ~YDYVFDW
NCU00685.1	KMTTPTEVLCRGFPNEFAIYLNYTRSLRFDDKPDYSYLRKIFRDLFVREGFH2QPQPADS
FG08731.1 	KMTTPTEVLCRGFPNEFAIYLNYTRSLRFDDKPDYSYLRKIFRDLFVREGFQ~YDYVFDW
AN4563.1  	KMTTPTEVLCRGFPNEFSIYLNYTRSLRFDDKPDYSYLRKIFRDLFVRESFQ~YDYVFDW
          	*****************:*******************************.*:      * 

MG02829.1 	T0DPSQQAQPGTVAQSS--------------------------RP------RNKNLIAG-
NCU00685.1	K~EAAQAAARKDPQQALR--------------------------------SGSRKPMTG-
FG08731.1 	T~VYKYQKNAQAIAQAAGQANPEDDEKARANRTNAATAGQSAAKPNAIPSTRRKMLERGS
AN4563.1  	T0KDKEAEEQARRQGATMPLTTSG-----------------AAKPGAISSQRRKIMERG-
          	.              :    ....                 :: ..: .:   :    * 

MG02829.1 	---EDTPNTRGAVGGSDRM~-------------------
NCU00685.1	----ETPETSRAVGGSDRM~-------------------
FG08731.1 	GAGVDTPDTNRAIGGSDRM~-------------------
AN4563.1  	-ALDNTPDTNRAVGGSDRI2HRGFPQLVGSVDFQVYVRR
          	 :  :**:*  *:*****:   . ..  .: . .
```
